# A Bibliometric Analysis of Global Scientific Research on Scrub Typhus

**DOI:** 10.1155/2020/5737893

**Published:** 2020-10-16

**Authors:** Taha Hussein Musa, Tauseef Ahmad, Wei Li, Joseph Kawuki, Mohammed Nasiru Wana, Hassan Hussein Musa, Pingmin Wei

**Affiliations:** ^1^Key Laboratory of Environmental Medicine Engineering, Ministry of Education, Department of Epidemiology and Health Statistics, School of Public Health, Southeast University, Nanjing, 210009 Jiangsu Province, China; ^2^Biomedical Research Institute, Darfur College, Nyala, Sudan; ^3^Key Laboratory of Environmental Medicine Engineering, Ministry of Education, Global Health, School of Public Health, Southeast University, Nanjing, 210009 Jiangsu Province, China; ^4^Department of Biological Sciences, Faculty of Science, Abubkar Tafawa Balewa University, Bauchi-740272, Nigeria; ^5^Department of Medical Microbiology, Faculty of Medical Laboratory Sciences, University of Khartoum, Khartoum, Sudan

## Abstract

**Objective:**

The rise of zoonotic diseases has become a global health issue around the world. The present study is aimed at assessing the global status and the trends in scrub typhus (ST) research.

**Methods:**

Publications related to ST studies from 1945 to 21^st^ July 2020 were retrieved from the Scopus database. The search for the ST literature was conducted using the entry terms of the MeSH (Medical Subject Headings) database. Full research articles and reviews were included in the analysis, and no limitation to the language was specified. Key bibliometric indicator analysis was performed using Microsoft Excel, Bibliometrix (an R package), GraphPad Prism 5, and VOSviewer (version 1.6.6).

**Results:**

A total of 1567 publications were retrieved. The results revealed a significant increase in the number of ST publications over time. The documents received an average of 11.22 citations per document. Mahidol University in Thailand (258, 16.46%) was the most productive institution, while the *American Journal of Tropical Medicine and Hygiene* published the most ST articles (88, 5.62%). Korea (195, 12.44%) was the most productive country, followed by India (178, 11.36%) and China (106, 6.76%). Richards AL was the most productive author with 36 articles.

**Conclusions:**

The study findings provide useful insights into the global efforts and works related to the progress of ST research, which can be used to identify future research areas, such as vaccine development.

## 1. Introduction

Scrub typhus (ST) is a vector-borne disease carried by the chigger mite [1]. It is endemic to numerous countries in the Asia-Pacific region [2]. It is considered the most critical vector-borne infection associated with travelers [3, 4] and has a higher incidence rate among the farmers than the nonfarmers in rural areas [5]. In addition, ST is a significant health issue among military personnel [6].

Up to date, more than one billion people worldwide are at risk of ST, and one million cases occur annually, especially in areas with limited access to health care services [7], which needs to be targeted via public health interventions [5]. The epidemiology of the disease traces back to World War II [8], and it is reemerging in many Southeast Asian countries [7]. Previously, it was endemic in a specified area mainly known as the “Tsutsugamushi Triangle” [2]. However, it is no longer restricted to the Tsutsugamushi Triangle [9].

In the past years, there have been significant scientific revolutions in ST research, specifically in fields that identify the ST patients' complications, treatment, diagnosis, and prevention [10, 11]. Thus, many documents have focused on the genotypic diversity of *O. tsutsugamushi* in the world, promising potential resources for diagnosis as well as the development of vaccines [2, 12, 13].

Therefore, we employed the concept of bibliometric analysis as a tool that uses scientific databases to draw relationships among academic journal citations and deduce trends and research directions within specific topics. The concept provides a better understanding of the aspects of science through systematic analysis of authors and journal citation reports [14, 15]. This bibliometric analysis, therefore, is aimed at assessing the global status and the trends in scrub typhus (ST) research, from 1945 to 2020.

The results of this analysis could help not only to provide an overview of ST publications based on predefined criteria (such as countries/region, institutions, productive authors, and journals, among others) but also assess the characteristics of ST publications.

## 2. Material and Methods

### 2.1. Data Sources

The data used in this study was obtained from the Scopus database (http://www.scopus.com/) through a comprehensive online search of ST documents published between 1945 and 2020.

### 2.2. Search Strategy

**Figure 1:**
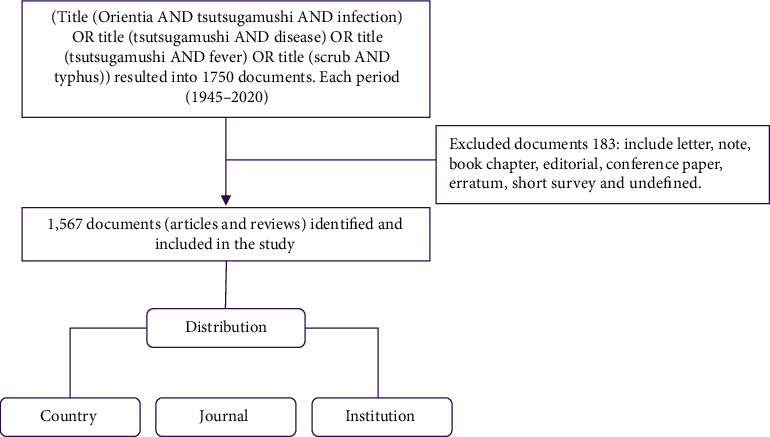
The inclusion and exclusion process of ST research.

All the ST-related publications from Scopus were collected on a single day (on 21^st^ July 2020) to avoid the daily update of the database. Moreover, the Scopus database provides the highest scientific quality of publication in comparison with other databases [14, 15]. In our study, an extensive search was done using Medical Subject Headings (MeSH), which imposes uniformity and consistency to the indexing of the biomedical literature (https://meshb.nlm.nih.gov/search). We used the search terms as follows: title (Orientia AND tsutsugamushi AND infection) OR title (tsutsugamushi AND disease) OR title (tsutsugamushi AND fever) OR title (scrub AND typhus), as shown in Figure 1. Regarding manuscript types, the search was restricted to only “article” and “review.” Two reviewers (THM and JK) independently screened the title to compile a list of the top 10 most-cited articles of scrub typhus. There is no ethical approval required for this study since there was no direct contact with human or animal subjects involved. Finally, the resulting bib.txt data was downloaded from Scopus, and as a result, 1567 publications related to ST were the subject of further analysis.

### 2.3. Data Collection

The following data were extracted from the retrieved publications: title, year of publication, author details, country, institution, journal, and number of citations, among others. This was used to evaluate citation density (i.e., an average of the number of citations received per year and per article), annual trend of publications, most productive authors, institutions and journals, and country contributions.

### 2.4. Data Analysis and Data Visualization

The key bibliometric indicators were analyzed using Bibliometrix, an R package, and these included the following: publication trends, citation scores, top 10 authors, journals, institutions, funding agencies, and cooccurrence of keywords, among others. GraphPad Prism 5 was used for time trend statistical analysis [16]. Map construction, cooccurrence network analysis, and visualization were conducted using the VOSviewer (version 1.6.6) package program (Leiden University, Leiden, The Netherlands). The program is freely available (https://www.vosviewer.com).

## 3. Results

### 3.1. Publication Output and Document Types

From the search criteria, a total of 1567 ST documents were identified, of which research articles dominated (1499, 95.66%) followed by review papers (68, 4.34%). When assessing the number of publications by language, most were published in English (1337, 85.32%), followed by Japanese (122, 7.78%) and Chinese (51, 3.25%), while other languages included Russian, Korean, Dutch, German, Italian, and Spanish, among others (57, 3.64%).

### 3.2. Annual Publication Trends and Citations

**Figure 2:**
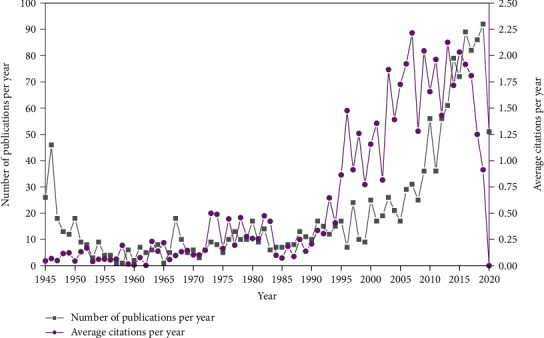
Global publication and citation trend in ST research from 1945 to 21^st^ July 2020.

The annual growth trends of publications and citations regarding ST are shown in Figure 2. There is a steady increase in publications by year, with a notably higher output in the last 20 years; we can see that (*n* = 25; 1.60%) in 2000 to (*n* = 56; 3.57%) in 2010, to (*n* = 72; 4.59%) in 2015, and to (*n* = 92; 5.87%) in 2019. Similarly, average citations per year (number of citations in a given year divided by total documents in that year) followed almost the same trend. The increase in the amount of the papers and the average citations per year were statistically significant (*P* < 0.001). The retrieved publications received an average of 11.22 citations per document.

### 3.3. Characteristics of the Top 10 Cited Papers

Table 1 shows the top 10 papers cited in ST research ordered by the total number of citations. The top 10 papers contributed to 1596 of the total citation scores. Among the top 10 articles, 5 articles were published as literature reviews and 5 as full research articles. The article by Watt and Parola [17] published in 2003 entitled “Scrub Typhus and Tropical Rickettsioses” was the most cited (*n* = 254 citations) and was published as a literature review. Of the top 10 highly cited articles, two [2, 18] were published in the journal of *Clinical Infectious Diseases*, which holds an impact factor IF = 8.313, and one [10] in *Lancet* with an impact factor of IF = 60.392.

### 3.4. Top 10 Most Productive Authors and Most Productive Institutions

A total of 4616 authors contributed to the total number of ST publications. The top productive authors are listed in Table 2, of which Richards AL ranked first with 43 publications, followed by Paris DH with 33 publications, Blacksell SD with 31 publications, and Kim D-M with 29 publications.

Regarding the most productive institutions, 904 institutions were identified. Mahidol University in Thailand was the leading research institution with 258 (16.46%) documents, followed by the Christian Medical College in India (197, 12.57%), Seoul National University College of Medicine in Seoul, South Korea (92, 5.87%), Naval Medical Research Center in the United States (81, 5.17%), and Institute for Medical Research in Malaysia (72, 4.59%). Among the top ten institutions, four are located in India, two in South Korea, one in China, and one in Thailand. We found that Southeast Asian institutions are the most active in the field of ST research.

### 3.5. Most Productive Journals

**Figure 3:**
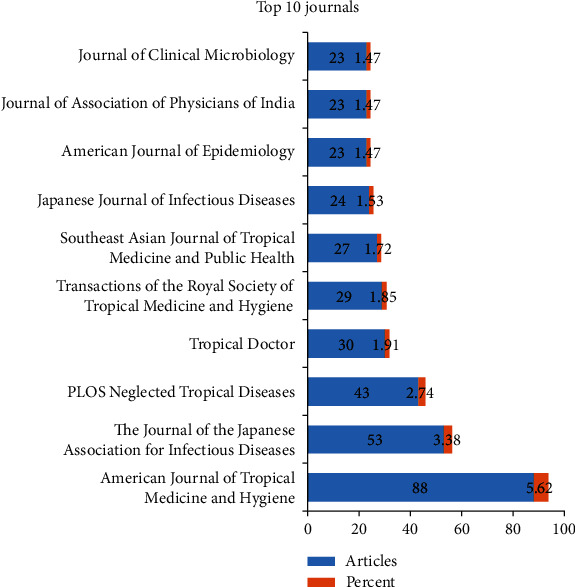
The distribution of the top 10 popular Journals.

Of 491 journal sources of ST publications, only the top 10 most active journals in the field are presented in Figure 3. Based on our Scopus data, the most productive journal was the *American Journal of Tropical Medicine and Hygiene* with 88 (5.62%) documents, followed by the *Journal of the Japanese Association for Infectious Diseases* (53, 3.38%) and PLOS Neglected Tropical Diseases (43, 2.74%), among others.

### 3.6. Most Productive Countries

Table 3 shows the top 10 productive countries in publishing research related to ST. Korea published the greatest number of documents (195, 12.44%), followed by India (178, 11.36%), China (106, 6.76%), Japan (67, 4.28%), Thailand (66, 4.21%), the USA (63, 4.02%), Malaysia (15, 0.96%), and Australia (12, 0.77%). When countries were ranked based on multiple country publications (MCP), only three countries had a high percentage of articles with international authors (*n* ≥ 15). This included Thailand (31, 1.97%), followed by the USA (27, 1.71%) and China (19, 1.21%). Publications from Korea (2438) had the highest citation score, followed by India (2291), Thailand (2138), and China (1943).

### 3.7. Funding Agencies

A total of 163 funding agencies were acknowledged. We found that the National Research Foundation of Korea is the most prominent funding agency (18, 1.15%), followed by the Wellcome Trust (14, 0.89%), National Natural Science Foundation of China (12, 0.77%), and Indian Council of Medical Research (12, 0.77) as shown in Table 4.

### 3.8. Most Frequent Keywords

Table 5 shows the top 10 keywords plus and author keywords that appeared most frequently in ST publications. The most commonly encountered author keywords in the retrieved literature were scrub typhus (468), *Orientia tsutsugamushi* (185), eschar (61), and tsutsugamushi disease (43). On the contrary, keywords plus are more general and included scrub typhus (2838), *Orientia tsutsugamushi* (1299), human (1214), article (1212), males (1127), female (1110), and adults (913).

### 3.9. Coauthorship Analysis and Collaboration between the Countries

**Figure 4:**
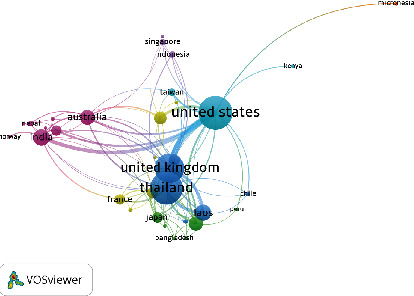
Coauthorship network of the top countries based on the total link strength (TLS).

The coauthorship network is presented in Figure 4 using VOSviewer. A threshold of two links (collaborations) was applied, which resulted in a total of 34 countries meeting the criteria. Based on the total citations (TC) and total link strength (TLS), the United States (TC = 4012, TLS = 126), Thailand (TC = 3355, TLS = 122), the United Kingdom (TC = 1278, TLS = 102), India (TC = 2656, TLS = 39), Laos (TC = 478, TLS = 37), Australia (TC = 487, TLS = 32), China (TC = 669, TLS = 25), Vietnam (TC = 142, TLS = 21), and Japan (TC = 650, TLS = 19) were the top 10 most collaborative countries in terms of ST research.

### 3.10. Coauthorship Analysis and Most Productive Authors

**Figure 5:**
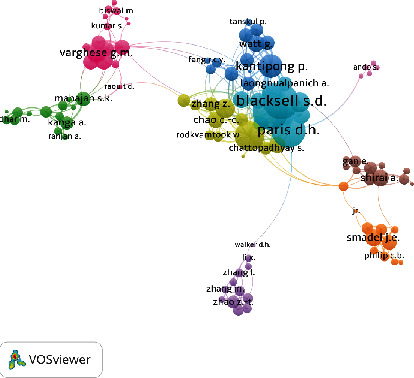
Coauthorship network of the most productive authors based on the total link strength (TLS).

Figure 5 shows the network visualization between the coauthors of ST research. A threshold of 5 authors was applied, which resulted in a total of 230 authors that met the threshold. The bubble size reflects the total number of citations. The five authors with the largest total link strength shown in the visualized analysis were Blacksell SD (TC = 907, TLS = 122), Day NPJ (TC = 712, TLS = 119), Paris DH (TC = 1094, TLS = 106), Richards AL (TC = 372, TLS = 64), and Kantipong P (TC = 466, TLS = 60).

### 3.11. Coauthorship Network between the Top Organizations

**Figure 6:**
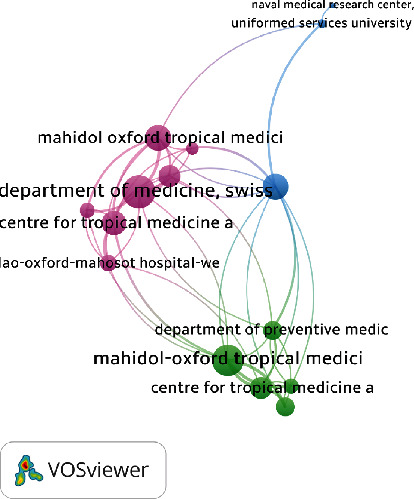
Coauthorship network and organizations based on the total number of reported documents of an organization.

Figure 6 shows the most collaborative institutions of ST research. A minimum of 3 organizations was set, and 75 organizations met the threshold. The most collaborative organizations included the following: the Department of Medicine, Swiss (TC = 71, TLS = 17), Mahidol-Oxford Tropical Medicine (TC = 321, TLS = 16), and Center for Tropical Medicine (TC = 159, TLS = 12) among others.

### 3.12. Geographical Distribution of Publications

**Figure 7:**
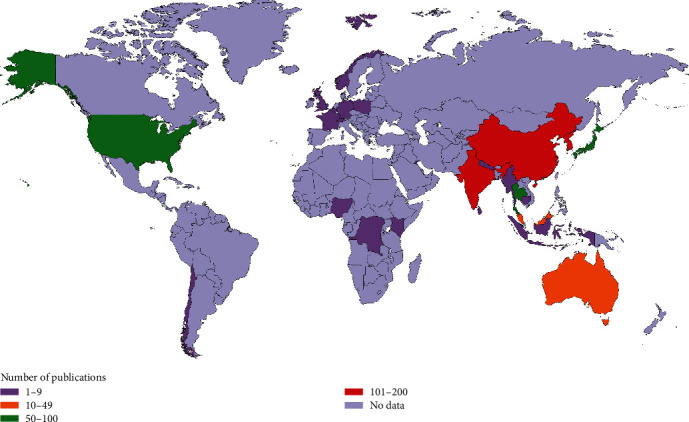
Geographical distribution of ST publications. The map was created using the mapchart.net program.

The geographical distribution of the retrieved ST documents is presented in Figure 7. Most published documents were from the Asia-Pacific region (red color). The geographic mapping shows that Korea (*n* = 195), India (*n* = 178), and China (*n* = 106) were the most significant producers of ST publications.

## 4. Discussion

Scrub typhus research has expansively increased during the last ten years, and the rapid growth in seroepidemiological data of *Orientia tsutsugamushi* infection has been reported from the area of the Tsutsugamushi Triangle [2, 9]. The growth of ST publications could be attributed to several factors like the emergence of new ST cases around the world [2, 5, 9], amidst no licensed vaccines to date nor effective vector control efforts across the world [19]. Despite the international efforts in reducing the risk factors and increasing awareness in endemic regions, the public health burden and global distribution of ST remain poorly known in the world [19]. Furthermore, ST is an unrecognized zoonotic disease with a significant burden on public health and economic status [20].

The present study shows that the earliest record was published in 1945 and discussed the epidemiology and control of ST disease that occurred during the Second World War [21]. Since 1985, the number of scholarly published ST documents has increased steadily [3, 4, 8, 12, 13], and the use of molecular epidemiology and genetic characterization techniques [22–24] has led to a better understanding of the research gap in *Orientia tsutsugamushi*. ST is a global public health problem within the area of the Asia-Pacific region, which can cause extensive endemic problems for people living in India, Korea, Taiwan, Sri Lanka, the Philippines, Thailand, and China [3, 7, 10, 12, 22].

There are more than 1567 ST articles that have been published in the 491 most prolific journals indexed in the Scopus database. These reported journals have created a scientific forum for researchers to discuss the hot themes in the field of ST. The involvement of these journals is a sign of the importance of ST topics to the scientific community in the world.

Analysis of the most productive authors and countries enables researchers to point out the link strength between the documents published and identify future research trends basing on coauthorship, productive authors, and organizations.

The top 10 most-cited documents were published in medical journals. A large impact factor of journals indicates that the articles in the journal obtain, on average, a more significant number of citations and top-cited papers usually appear in journals with high impact factors [25].

The analysis of the top-cited articles indicated that ST research covered various key aspects such as ST geographical distributions and genetic diversity, serological diagnostic, clinical image and disease complication, and ecology of chigger-borne infections, among others. Notably, the two top-cited documents by Watt and Parola [17] and Kelly et al. [2] both describe ST as a significant public health problem in tropical regions. They also present a clear picture of the geographical distribution and genetic diversity of the disease [2, 17]. Thus, the brief overview of the top 10 ST documents highlighted more insights into the research directions that highlighted hotspots and promising avenues for future research work in the ST field.

Furthermore, from the evidence of the geographical distribution map, it indicated that ST cases and research production were not restricted to the Tsutsugamushi Triangle [2]. The emergence of ST pathogens and scientific research production included other countries in the world [9, 11]. From the results, we can see that several African countries have also published a significant number of ST publications. However, their contribution was lesser compared to other countries, which could be attributed to a limited number of resources and funds to support publication fees in high-quality journals or databases.

In our study, coauthorship analysis was utilized to evaluate the cooperation between different institutions and authors. Results with higher total link strength indicate a greater degree of collaboration among institutions and authors in publishing and carrying out the ST research.

In addition, the keyword analysis highlighted the dominance of ST topics with keywords such as “human,” “male,” “female,” “middle-aged,” and “children,” among others. This gives vital information about the dynamics of the disease as well as the broader scope of ST research. However, key areas such as vaccine development and clinical trials were not reflected in the keyword occurrence. This implies more research efforts towards effective prevention alternatives like vaccines to address ST.

## 5. Strength and Limitations

This study is the first bibliometric analysis that evaluates and visualizes the trend of published ST documents from Scopus databases. Nevertheless, the study also has some specific limitations. We restricted our search to Scopus databases, and we did not compare our findings with other scientific databases such as Web of Science (WoS), Embase, Cochrane Library, PubMed, and Google Scholar, which could help provide a better overview of the ST-related published literature.

## 6. Conclusion

Our bibliometric analysis provides historical insights and perspectives of ST research during the past 75 years. Korea, India, and China were identified as the major contributors and play leading roles in the global ST research production. The *American Journal of Tropical Medicine and Hygiene* published the most significant number of articles on ST. Papers from the USA, India, Thailand, and Korea are most likely to have high citation scores. However, the study highlights a need for more focus and research on vaccine development to address this public health issue. Nevertheless, our findings may help to inspire future works in ST research and specifically encourage stakeholders, researchers, and institutions to focus on the identified hotspot fields.

## Figures and Tables

**Table 1:** The top 10 highly cited documents on ST.

Rank	Author, year	Document title	Journal	TC	TCY	TD	IF (2020)
1^st^	Watt G & Parola P, 2003	Scrub typhus and tropical rickettsioses	Current Opinion in Infectious Diseases	254	14.11	Review	4.557
2^nd^	Kelly DJ et al., 2009	Scrub typhus: the geographic distribution of phenotypic and genotypic variants of *Orientia tsutsugamushi*	Clinical Infectious Diseases	228	19.00	Article	8.313
3^rd^	Watt G et al., 1996	Scrub typhus infections poorly responsive to antibiotics in northern Thailand	Lancet	187	7.48	Article	60.392
4^th^	Seong SY et al., 2001	Orientia tsutsugamushi infection: overview and immune responses	Microbes and Infection	170	8.50	Review	2.373
5^th^	Traub R & Wisseman Jr CL, 1974	The ecology of chigger borne rickettsiosis (scrub typhus)	Journal of Medical Entomology	132	2.81	Article	1.955
6^th^	KOH GCHW et al., 2010	Review: diagnosis of scrub typhus	American Journal of Tropical Medicine and Hygiene	126	11.45	Review	2.126
7^th^	Berman SJ & Kundin WD, 1973	Scrub typhus in South Vietnam. A study of 87 cases	Annals of Internal Medicine	126	2.63	Article	21.31
8^th^	Blacksell SD et al., 2007	Scrub typhus serologic testing with the indirect immunofluorescence method as a diagnostic gold standard: a lack of consensus leads to a lot of confusion	Clinical Infectious Diseases	125	8.93	Review	8.313
9^th^	Tsay RW & Chang FY, 1998	Serious complications in scrub typhus	Journal of Microbiology, Immunology, and Infection	125	5.43	Article	3.493
10^th^	Yeon JJ et al., 2007	Scrub typhus: clinical, pathologic, and imaging findings	Radiographics	123	8.79	Review	4.967
	Total			1596	98.13		

TC: total citations; TCY: total citations per year; TD: type of document; IF: impact factor of journals (2020).

**Table 2:** Top 10 prolific authors and institutions.

Top ten authors	NP	TC	*h*_index∗	Top ten institutions, country	NP	%
Richards AL	43	986	18	Mahidol University, Thailand	258	16.46
Paris DH	33	1094	19	Christian Medical College, India	197	12.57
Blacksell SD	31	907	19	Seoul National University College of Medicine, Seoul, South Korea	92	5.87
Day NPJ	30	712	16	Naval Medical Research Center, United States	81	5.17
Kim D-M	29	502	11	Institute for Medical Research, Malaysia	72	4.59
Smadel JE	23	309	11	Chosun University, Gwangju, South Korea	57	3.64
Lee C-S	18	147	6	India Gandhi Medical College, Vellore, India	53	3.38
Osterman JV	18	369	12	All India Institute of Medical Sciences, India	47	3.00
Shirai A	18	409	9	Manipal University, India	44	2.81
Traub R	18	356	9	Shandong University, China	44	2.81

TC: total citations; NP: number of published documents by authors and institutions; *h*-index for the period 1945 to 21^st^ July 2020.

**Table 3:** Top ten most productive countries in publishing ST articles (1945-21^st^ July 2020).

Rank	Country	TPC (%)	TC	SCP (%)	MCP (%)
1^st^	Korea	195 (12.44)	2438	188 (12.00)	7 (0.44)
2^nd^	India	178 (11.36)	2291	165 (10.54)	13 (0.82)
3^rd^	China	106 (6.76)	1510	87 (5.55)	19 (1.21)
4^th^	Japan	67 (4.28)	606	62 (3.96)	5 (0.32)
5^th^	Thailand	66 (4.21)	2138	35 (2.23)	31 (1.97)
6^th^	USA	63 (4.02)	1828	36 (2.30)	27 (1.71)
7^th^	Malaysia	15 (0.96)	342	15 (0.95)	0 (0.00)
8^th^	Australia	12 (0.77)	194	7 (0.45)	5 (0.32)
9^th^	France	9 (0.57)	206	4 (0.26)	5 (0.32)
10^st^	Sri Lanka	6 (0.38)	94	5 (0.32)	1 (0.06)

TC: total citations; TPC: total number of publications by the corresponding author country; SCP: single country publications (intracountry publications); MCP: multiple country publications (intercountry publications).

**Table 4:** Top 10 funding agencies in scrub typhus research.

Rank	Funding sponsor (*n* = 163)	Documents (%)
1^st^	National Research Foundation of Korea	18 (1.15)
2^nd^	Wellcome Trust	14 (0.89)
3^rd^	National Natural Science Foundation of China	12 (0.77)
4^th^	Indian Council of Medical Research	12 (0.77)
5^th^	Korea Centers for Disease Control and Prevention	7 (0.45)
6^th^	Ministry of Health and Welfare	7 (0.45)
7^th^	Korea Health Industry Development Institute	6 (0.38)
8^th^	National Institutes of Health	6 (0.38)
9^th^	Chosun University	4 (0.26)
10^th^	Chonbuk National University Hospital	4 (0.26)

**Table 5:** Top 10 most frequently used author keywords and keywords plus.

Rank	Keywords plus		Author keywords	
	Terms	Frequency	Terms	Frequency
1^st^	Scrub typhus	2838	Scrub typhus	468
2^nd^	*Orientia tsutsugamushi*	1299	*Orientia tsutsugamushi*	185
3^rd^	Human	1214	Eschar	61
4^th^	Article	1212	Tsutsugamushi disease	43
5^th^	Male	1127	Rickettsia	37
6^th^	Female	1110	Children	28
7^th^	Adult	913	Doxycycline	25
8^th^	Humans	735	Elisa	18
9^th^	Middle-aged	597	India	18
10^th^	Aged	558	Weil-Felix test	18

## Data Availability

The data used to support the findings of this study are included within the article.
